# Genetic etiology of autism spectrum disorder in the African population: a scoping review

**DOI:** 10.3389/fgene.2024.1431093

**Published:** 2024-09-26

**Authors:** Olivier Hakizimana, Janvier Hitayezu, Jeanne P. Uyisenga, Hope Onohuean, Leonor Palmeira, Vincent Bours, Abdullateef Isiaka Alagbonsi, Annette Uwineza

**Affiliations:** ^1^ Department of Biochemistry, Molecular Biology and Genetics, School of Medicine and Pharmacy, College of Medicine and Health Sciences, University of Rwanda, Kigali, Rwanda; ^2^ Center for Human Genetics, Centre Hospitalier Universitaire Sart-Tilman, University of Liege, Liege, Belgium; ^3^ Department of Pediatrics, University Teaching Hospital of Kigali (CHUK), Kigali, Rwanda; ^4^ Department of Biology, College of Science and Technology, University of Rwanda, Kigali, Rwanda; ^5^ Biopharmaceutics Unit, Department of Pharmacology and Toxicology, School of Pharmacy, Kampala International University, Bushenyi, Uganda; ^6^ Department of Physiology, School of Medicine and Pharmacy, College of Medicine and Health Sciences, University of Rwanda, Kigali, Rwanda

**Keywords:** austism spectrum disorders, ASD, gene, genetics, genome, sequencing, africa

## Abstract

**Background:**

Autism spectrum disorder (ASD) is a neurodevelopmental disorder (NDD) characterized by significant impairments in social, communicative, and behavioral abilities. However, only a limited number of studies address the genetic basis of ASD in the African population. This study aims to document the genes associated with ASD in Africa and the techniques used to identify them. Additionally, genes identified elsewhere but not yet in Africa are also noted.

**Methods:**

Online databases such as Wiley Online Library, PubMed, and Africa Journal Online were used. The review was conducted using the keyword related to genetic and genomic ASD study in the African population.

**Result:**

In this scoping review, 40 genetic studies on ASD in Africa were reviewed. The Egyptian and South African populations were the most studied, with 25 and 5 studies, respectively. Countries with fewer studies included Tunisia (4), East African countries (3), Libya (1), Nigeria (1), and Morocco (1). Some 61 genes responsible for ASD were identified in the African population: 26 were identified using a polymerase chain reaction (PCR)-based method, 22 were identified using sequencing technologies, and 12 genes and one *de novo* chromosomal aberration were identified through other techniques. No African study identified any ASD gene with genome-wide association studies (GWAS). Notably, at least 20 ASD risk genes reported in non-African countries were yet to be confirmed in Africa’s population.

**Conclusion:**

There are insufficient genetic studies on ASD in the African population, with sample size being a major limitation in most genetic association studies, leading to inconclusive results. Thus, there is a need to conduct more studies with large sample sizes to identify other genes associated with ASD in Africa’s population using high-throughput sequencing technology.

## 1 Introduction

Autism spectrum disorder (ASD), a widespread and clinically heterogeneous neurodevelopmental disease (NDD), is defined by irregularities in social interactions and repetitive or limited behavioral patterns (Hodges et al., 2020) that manifest in early postnatal life. ASD shows various symptoms and severities which significantly burden affected individuals, caregivers, and families (van Heijst and Geurts, 2015). The World Health Organization (WHO) reported ASD as a major worldwide public health issue in underdeveloped nations (World Health Organization, 2013), being among the leading causes of disability in children (Salari et al., 2022). Globally, one in a hundred children have ASD, indicating an increase in the disorder’s prevalence (Zeidan et al., 2022). Although the prevalence of ASD in low- or middle-income countries, including in Africa, is controversial, this disease burden has recently been found to be underestimated (Fombonne et al., 2021; Aderinto et al., 2023). According to a recent global systematic review and meta-analysis, the prevalence of ASD was 0.4% (95% CI: 0.1–1) in Asia, 1% (95% CI: 0.8–1.1) in America, 0.5% (95% CI: 0.2–1) in Europe, 1% (95% CI: 0.3–3.1) in Africa, and 1.7% (95% CI: 0.5–6.1) in Australia (Salari et al., 2022).

With a 50%–90% heritability estimate, ASD is a neuropsychiatric condition that is complicated and genetically heterogeneous (Kainer et al., 2023), although *de novo* gene variations also have an outstanding contribution. To date, over 200 susceptible genes have been identified as being linked to ASD. Many genes have been found to be the most prevalent risk factors for the development of ASD, including *MTHFR*, *RELN*, *CACNA1C*, *SHANK*, and *VDR* (Wiśniowiecka-Kowalnik and Nowakowska, 2019; Wei et al., 2021). Study findings have shown several chromosome regions, including one or more with susceptible genes for autism (Monaco and Bailey, 2001). Moreover, several studies have shown that prenatal, perinatal, and postnatal environmental factors are associated with ASD (Gardener et al., 2011). According to some reports, factors such as medications, chemical exposures, parental age, nutrition, and prenatal environment can account for up to 40%–50% of the variation in ASD liability (Gaugler et al., 2014; Deng et al., 2015). ASD development, however, may also be influenced by the interplay of genetic and environmental factors, as suggested by Tordjman et al. (2014) and Santos et al. (2022). For instance, oxidative stress may be a potential mechanism in genetic disorders linked to ASD that connect genetic and environmental factors. Fragile X messenger ribonucleoprotein (FMRP), primarily involved in mRNA binding, is absent in *FXS* due to a loss of *FMR1* expression. As demonstrated in *FMR1*-knockout mice, the lack of FMRP increases oxidative stress (De Diego-Otero et al., 2009). Any chromosome or gene changes that lead to an increase in oxidative stress could potentially be a factor in the manifestation of a behavioral autistic phenotype. In addition, the association of particulate matter with an aerodynamic diameter of 10 μm or less (PM10) and cognitive neurodevelopment has been found to be significantly mediated by DNAm; CpG sites mapped several genes, including DYRK1A, which has shown lower expression in South African ASD patients (Feil et al., 2023).

African populations are the most genetically varied in the world, having three times more uncommon variants than in Europe and East Asia (1,000 Genomes Project Consortium et al., 2012); they are exploited to find genes linked to diseases (Gomez et al., 2014). African genomes offer a unique resource for identifying new genetic loci and for very effective genetic fine-mapping due to their extensive genetic diversity and low linkage disequilibrium (Tishkoff and Verrelli, 2003; Campbell and Tishkoff, 2008). Despite Africa’s high human genome diversity, few genes or single nucleotide polymorphisms (SNPs) of potential ASD risk genes have been discovered in that population. This results from (i) no validated tools available for the diagnosis of ASD in the African population, (ii) genetic studies previously conducted in African countries used a small sample size, or (iii) most studies not using high throughput sequence technology for analysis like in the two Egyptian studies (Salem et al., 2013; Abdelrahman et al., 2015).

The present scoping review aims to identify risk genes associated with ASD in the African population, while other ASD-associated genes reported from other populations but not yet identified in Africa will also be discussed.

## 2 Methodology

The Preferred Reporting Items for Systematic Reviews and Meta-Analyses extension for Scoping Reviews (PRISMA-ScR) checklist was followed in this scoping review (Tricco et al., 2018). The selection included studies conducted in Africa that reported gene(s) or genetic variants associated with ASD, performed only on human beings or human cells, published from 1 January 2000 to 29 February 2024 and published in English. In addition, we excluded papers published as letters, in books, in gray literature, and reviews, as well as papers for research conducted using data from online databases. This scoping review followed Arksey and O'Malley’s scoping framework that proposed six stages of conduct: 1) specify the research question, 2) identify relevant literature, 3) select studies, 4) map out the data, 5) summarize, synthesize, and report the results, and 6) include expert consultation (Arksey and O’Malley, 2005). Electronic research was performed to identify relevant peer-reviewed articles mainly using the Wiley Online Library and PubMed databases. In order to identify genetic variants linked to ASD in Africa, the search approach included multiple sets of broad search terms with “AND” in each database. The search terms: “autism OR autistic OR Asperger Syndrome OR pervasive developmental disorder” were first used. Second, a group of search terms that combined important key terms, including “genetic study OR sequencing OR genomic study OR case/control study OR family-based study”, were employed. Lastly, a search specified the region as “Africa” and the names of individual African countries. We downloaded the details of all articles in the data collection sheet and categorized studies into research themes (genomic association studies, sequencing technology, and PCR-based methodology) and countries where the studies were conducted.

### 2.1 Article selection

Every research paper identified through the search strategy was exported into collections. Two authors independently examined the titles and abstracts of every article and then sequentially reviewed the full texts of all the articles identified for pertinent publications. The data-charting involved two authors: using a standardized form, the first author charted data from the selected publications, and the second verified the charted data. Study features such as first author, year of publication, country of study, population, sample size, age, genes or genetic variants identified, study design, body tissue type for sampling, and main findings were carefully extracted from every selected paper. Consistent with the scoping review process, the authors did not assess the selected publications for methodological quality or risk of bias. However, some excluded studies from the African continent were project theses. The abstracted data are presented in Supplementary File S1.

## 3 Result

We initially found 605 papers, including 558 articles from the Wiley Online Library and 47 articles from PubMed. Subsequently, we removed 528 articles: five were duplicates, and the remaining 523 lacked the necessary information for being a genetic study conducted in Africa, which is a key variable for this scoping review. Among the 77 articles screened, 67 were deemed irrelevant after screening their titles, abstracts, and full texts; they thus were excluded from the study. Ten relevant articles from the Wiley Online Library and PubMed were eventually included in this scoping review.

**FIGURE 1:**
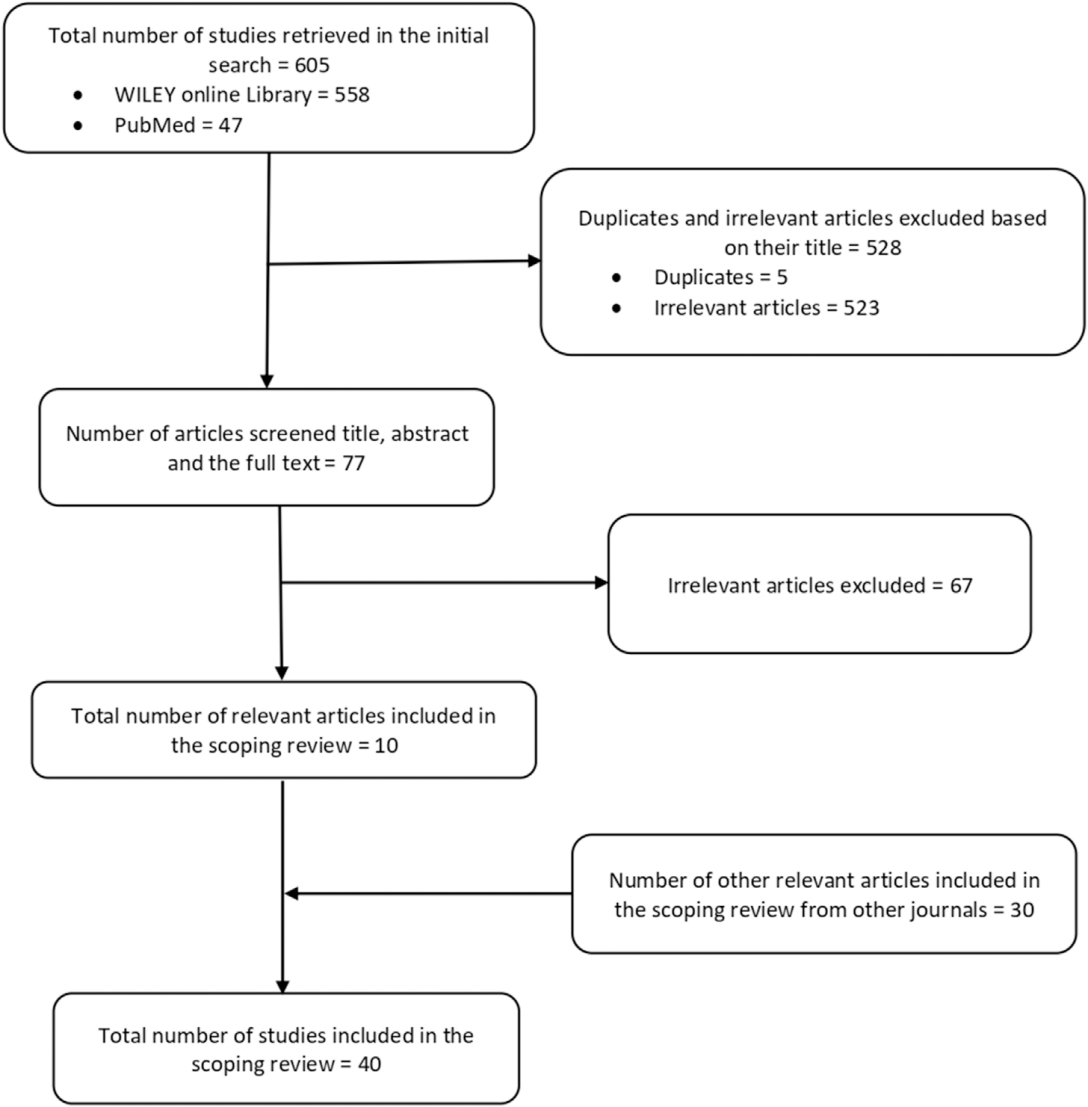
Diagram of the study selection process.

Additionally, 30 relevant articles identified by searching other journals in the Africa Journal Online (AJOL) were included in the study. There were no methodological issues that necessitated excluding any related ASD genetic study from Africa in this review. The 40 most pertinent articles (Supplementary File S1) used as source documents for this review were selected through these meticulous selection procedures, summarized in an adapted PRISMA-ScR model shown in Figure 1.

A total of 61 genes were identified in the African population as being responsible for ASD (Table 1). Among the 40 relevant studies, Egyptian and South African ethnicities were the most studied populations, appearing in 25 and 5 articles which represented 62.5% and 12.5%, respectively. Additionally, Tunisia (four articles; 10%), the East African population (Ethiopia, Eritrea, Kenya, and Somalia) (three articles; 7.5%), and the populations of Libya, Nigeria, and Morocco (one article each; 2.5%) (Table 2) were also studied. No other suitable publications were found dealing with the remaining African countries. Furthermore, 20 ASD risk genes identified in non-African populations have not been confirmed/reported in Africa (Table 3).

**TABLE 1:** List of ASD genes identified so far in Africa’s population.

NO	Gene	Country	Study design	Author	Genetic implication
1	AADAT	Egypt	PCR method	Higazi et al., 2021	Graves’ disease and thyroid disease
2	CACNA1C	East Africa (Ethiopian, Eritrea, and Kenya)	WGS	Tuncay et al., 2023	Timothy syndrome features
3	CHD7				CHARGE syndrome
4	COMT	Egypt	PCR method	Karam et al., 2013	Schizophrenia and ADHD
5	C4B	Egypt	PCR method	Mostafa and Shehab (2010)	Chronic hypertension, thromboses, and schizophrenia
6	DRD1	Egypt	PCR method	Azzam et al., 2018	Psychosis and schizophrenia
7	DRD2 A1+	Egypt	PCR method	Salem et al., 2013	Type 2 diabetes
8	DRD4	Egypt	PCR-RFLP	Kamal et al., 2015	ADHD
9	FEZF2	Morocco	WES	Bensaid et al., 2019	Uterine inversion and thymic dysplasia
10	FMR1	East Africa (Ethiopia, Eritrea, and Kenya)	WEG	Tuncay et al., 2023	ID and fragile X syndrome
11	GABA	Egypt	PCR method	El-Ansary et al., 2021	Neurodevelopmental disorders and epilepsy
12	GSTM1	Egypt	PCR method	Said et al., 2021	Urinary system cancer and metastatic breast cancer
13	GSTT1	Egypt	PCR method	Said et al., 2021	Metastatic breast cancer and sickle cell disease
14	HAAO	Egypt	PCR method	Higazi et al., 2021	Hypospadias
15	HLA-DRB1	Egypt	PCR method	Mostafa et al., 2012	Rheumatoid arthritis (RA)
16	HLA-DR4	Egypt	PCR method	El-Hossiny et al. (2023)	Rheumatoid arthritis (RA)
17	HTR1A	Egypt	PCR method	Yahya et al., 2019	Bipolar disorder
18	5-HTTLPR	South Africa	PCR method	Arieff et al., 2010	Anxiety and depression
19	5-HTTLPRS	Egypt	PCR method	Meguid et al., 2015	Anxiety and depression
20	5-HT2A	Egypt	PCR-RFLP	Abdelrahman et al. (2015)	Anxiety, depression, drug addiction, and schizophrenia
21	IL-1β-511 and IL-1RA	Egypt	PCR method	Saad et al., 2020	Gastric cancer and osteoporotic fractures
22	IL-12	Egypt	PCR method	Ibrahim et al., 2015	Atherosclerosis and coronary artery disease
23	MAOA	Egypt	PCR method	Higazi et al., 2021	Bipolar disorder, depression, and paranoid schizophrenia
24	PCDHAC1	East Africa (Ethiopia, Eritrea, and Kenya)	WES	Tuncay et al., 2023	Higher birth weight percentiles and larger newborn head circumferences
25	Q6NUR6	Tunisia	PCR method	Bayou et al., 2010	ASD
26	RELN	South Africa and Egypt	PCR method	Sharma et al., 2013, Abdelhady et al., 2022	Bipolar disease, schizophrenia, and autism
27	SATB2	Morocco	WES	Mounia et al., 2019	SAS
28	SBCADD	East Africa (Somalia, Eritrea, and Ethiopia	Sanger sequencing	Oivind et al., 2007	Developmental delay
29	SHANK2	Ethiopia	NGS	Lu et al., 2018	Mild-to-moderate ID
30	SHANK3	Egypt	PCR-RFLP	Meguid et al., 2020	Moderate-to-severe ID, severely impaired speech, schizophrenia, and mild dysmorphic features
31	TAMRTKs	Egypt	PCR method	Mostafa et al., 2022	Glioblastoma multiforme
32	TBC1D8	Libya	WES	Zeglam and Alhmadi, 2020	Independent prognosis factor for ovarian cancer
33	TBR1	Morocco	WES	Mounia et al., 2019	NDD combining features of ASD, ID, and speech delay
34	TCF7L2	East Africa (Ethiopia, Eritrea, and Kenya)	WGS	Tuncay et al., 2023	Type 2 diabetes
35	TSHZ3	Morocco	WES	Mounia et al., 2019	Autism, cognitive disabilities, and language disturbance
36	VDR	Egypt	PCR-RFLP	Arafa et al., 2021	Severity of autoimmune thyroid diseases; HT and GD
37	ERICH1	Tunisia	aCGH technology	Fethia et al., 2022>	Adenoma risk
38	CELF4				Seizures and neuroticism
39	CHRFAM7A				Schizophrenia and bipolar disease
40	FTHL17				Tumorigenesis
41	NEXMIF				X-linked intellectual disability, ASD, and epilepsy
42	NLGN4X				Intellectual disability, ASD, anxiety, and ADHD
43	PRKN				Parkinson’s disease
44	SPN				Tumor progression
45	SYCE3				Human infertility
46	UQCRC2				Lactic acidosis
Differential methylated genes					
47	PGC-1α	South Africa	NGS	Bam et al., 2021	Obesity, diabetes, neurodegeneration, and cardiomyopathy
48	STOML2				Pancreatic cancer
49	MFN2				Charcot–Marie–Tooth disease type 2A
50	FIS1				Diabetes
51	OPA1				Optic atrophy
52	GABPA				Hepatocellular carcinoma and bladder cancer
53	AHI1	South Africa	WG-Methylation	Stathopoulos et al. (2018)	Joubert syndrome and schizophrenia
54	GLRA2				X-linked neurodevelopmental disorders and high myopia
55	SETD5				ID, ASD, and KBG syndrome
56	MTR				Cardiovascular diseases, breast and prostate cancer, birth defects, and congenital anomalies
57	RTL	Egypt	PCR method	Salem et al., 2023	KOS14 and TS14
58	LINE-1				Schizophrenia, bipolar disorder, and major depressive disorder
59	PCCB	South Africa	Methylation analysis	Stathopoulos et al. (2020)	Propionic acidemia and biotinidase deficiency
60	PCDHA12				Developmental delay, movement disorder, epilepsy, microcephaly, and visual impairment
61	*De novo* balanced (7; 16) (p22.1; p16.2) translocation	Tunisia	Cytogenetics and FISH	Bayou et al. (2008)	Numerous genomic tests for autism have found chromosome 16p, most likely home to an autism-susceptibility variation

aCGH, micro-array-based comparative genomic hybridization; NGS, next-generation sequencing; WG, whole genome; WES, whole-exome sequencing; ADHD; attention-deficit hyperactivity disorder; ID, intellectual disability; KOS14, Kagami–Ogata syndrome; TS14, temple syndrome; HT, Hashimoto’s thyroiditis; GD, Graves’ disease; SAS, SATB2-associated syndrome.

**TABLE 2:** National distribution of identified genes associated with ASD in Africa.

Countries	No. of studies	%	N^0^ of genes associated with ASD
Egypt	25	62.5%	24
South Africa	5	12.5%	14
Tunisia	4	10%	12
East Africa (Ethiopia, Eritrea, Kenya, and Somalia)	3	7.5%	6
Morocco	1	2.5%	4
Libya	1	2.5%	1
Nigeria	1	2.5%	0

**TABLE 3:** List of ASD genes identified in non-African populations yet to be confirmed/reported in Africa.

No.	Gene	Country	Study design	Author
1	ASMT	China and Sweden	WES and PCR method	Wang et al., 2013; Jonson et al., 2014
2	CHD8	United States	Sanger sequencing	Bernier et al. (2014)
3	CNTNAP2	China and Brazil	PCR method	Li et al., 2010; Nascimento et al., 2015
4	DRD3	Netherlands and United Kingdom	SNP genotyping	De Krom et al. (2009)
5	EN2	China and India	PCR method	Wang et al., 2008, Sen et al., 2010
6	FOXP1	Taiwan and Canada	PCR method and Sanger sequencing	Chien et al., 2013; Hamdan et al., 2010
7	GABRB3	United States, United Kingdom, and Taiwan	PCR method and Sanger sequencing	Buxbaum et al., 2002; Warrier et al., 2013; Chen et al., 2014
8	GRIN2B	China and Korea	Sanger sequencing, PCR method	Pan et al., 2014; Hee Jeong et al., 2012
9	HoxA1	United States and Italy	Sanger sequencing and PCR method	Ingram et al., 2000; Conciatori et al., 2004
10	SRRM4	Italy and Japan	RNA sequencing and GWAS	Irimia et al., 2014; Narita et al., 2020
11	MECP2	United States and China	Sanger sequencing and WES	Nagarajan et al., 2006; Wen et al., 2017
12	MET	United States and Italy	PCR method	Campbell et al., 2009; Jackson et al., 2009
13	NLGN3	China and Italy	Sanger sequencing and WES	Xu et al., 2014; Oleari et al., 2023
14	NLGN4	France, Bulgaria, and Japan	PCR and sequencing	Laumonnier et al., 2004; Avdjieva-Tzavella et al., 2012; Toya et al., 2023
15	NRXN1	United States and China	GWAS and array CGH sequencing	Liu et al., 2012; Liu et al., 2012; Griswold et al., 2015
16	OXTR	China and United Kingdom	SNP genotyping	Wu et al., 2005; Di Napoli et al., 2014
17	PTCHD1	European, Caucasian	Sequencing	Torrico et al., 2014
18	PTEN	France, and United States	WES	Buxbaum et al., 2007, O’Roak et al., 2012
19	SLC25A12	United States and Finland	SNP genotyping	Ramoz et al., 2004, Joni et al., 2008
20	SYNGAP1	Canada	Sanger sequencing	Hamdan et al., 2011, Berryer et al., 2013

### 3.1 Genetic techniques adopted for identifying genetic variants linked to ASD in Africa

**FIGURE 2:**
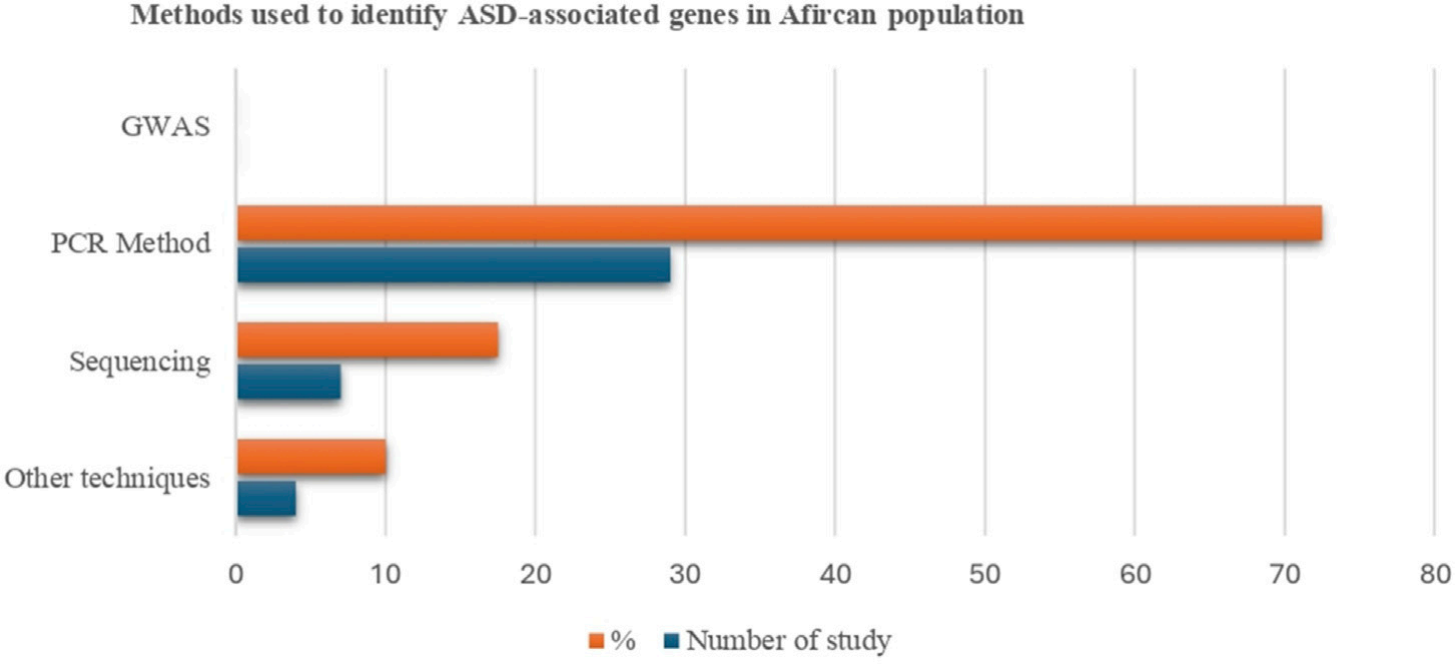
Selected articles on the association of genetic factors and ASD by study design.

We categorized the research into three main themes based on study design: GWAS (n = 0), sequencing-based technology (n = 7), and PCR-based methods (n = 29) (Figure 2. However, the remaining studies used other molecular biology techniques such as methylation analysis (n = 1), hydride generation technique (n = 1), microarray-based comparative genomic hybridization (aCGH) technology (n = 1), and cytogenetics and fluorescent *in situ* hybridization (FISH) analysis (n = 1). It was shown that different genetic techniques were performed for various studies, and numerous ASD-associated genes were identified. In the African population, no gene was found that utilized GWAS; 26 genes were identified using a PCR-based method, 22 genes, including 10 differentially methylated, were discovered using sequencing technologies, and 12 were found through other techniques. Except for GWAS, most research methods used globally for genetic studies on ASD have been applied in Africa. Our findings demonstrate that numerous genetic variations could contribute to the underlying genetic basis of ASD in African communities.

#### 3.1.1 Genome-wide association studies

Several developed countries have employed the GWAS technique to discover common and rare variants linked to ASD. However, no single GWAS research study was performed for the African population among our retrieved data.

#### 3.1.2 Sequencing technologies

Seven studies used sequencing technologies and identified 12 genes associated with ASD in the African population: *CACNA1C, CHD7, FEZF2*, *FMR1*, *PCDHAC1, SATB2, SBCADD*, *SHANK2*, *TBC1D8, TBR1, TCF7L2*, and *TSHZ3*. In addition, 10 differentially methylated genes—*AHI1*, *FIS1, GABPA, GLRA2*, *MFN2, MTR*, *OPA1, PGC*-*1α*, *SETD5*, and *STOML2*—were identified as linked to ASD by these technologies. A number of potentially pathogenic variations in known ASD genes—*CACNA1C*, *CHD7*, *FMR1*, *TCF7L2*, and *PCDHAC1*—were found to be novel autism susceptibility genes using whole genome sequencing (WGS) on an East African cohort to investigate the genetics of ASD (Tuncay et al., 2023). The results from this study suggested a higher prevalence of ASD in East African children and demonstrated the value of admixture analysis and African genetic diversity in understanding the etiology of complex disorders.

Several studies using next-generation sequencing (NGS) and identifying DNA methylation as causing ASD have been conducted in South Africa. Whole-genome DNA methylation screening showed that *GLRA2* and *AHI* were differentially methylated in children with ASD from South Africa (Stathopoulos et al., 2020). Moreover, it was shown that the DNA methylation-related genes *SETD5* and *MTR* were differentially methylated, linking them to ASD (Stathopoulos et al., 2018). Several genes including, *PGC*-1*α*, *STOML2*, *MFN2*, *FIS1*, *OPA1* and *GABPA*, have different levels of methylation related to mitochondrial biogenesis, fission, and fusion in ASD, according to next-generation bisulfite sequencing technology (*p* < 0.05) (Bam et al., 2021). Furthermore, this South African study showed the correlation between methylation at *PGC*-*1α* and metabolomic evidence of mitochondrial malfunction. The results of another South African ASD cohort showed that methylation levels of case and control for *PCCB* and *PCDHA12* varied widely from 9%–49% and 0%–54%, respectively. Further evidence that DNA methylation is a crucial epigenetic component in the pathogenesis of ASD comes from the hypothesis that the differentially methylated genes linked to ASD in this cohort have a functional role in mitochondrial homeostasis and dysregulation.

A previous study showed a mutation in the SBCADD gene among people originating from Somalia and Eritrea. This finding was repeated in a four-year-old autistic Somali boy (Kanavin et al., 2007), suggesting that this mutation is relatively common in this demographic.

#### 3.1.3 PCR-based methods

A total of 29 ASD genetic studies in Africa were conducted using PCR-based approaches such as polymerase chain reaction–restriction fragment length polymorphism (PCR-RFLP) and real-time PCR. However, these methods identified 26 genes: *AADAT, C4B, COMT* Val58Met, *DRD1*, *DRD2A1+, DRD4, GABA, GSTM1, GSTT1, HAAO, HLA-DRB1, HLA-DR4, HTR1A*, *5-HTTLPR,* 5-*HTTLPRS*, 5-*HT2A, IL-1β*-*511* and *IL*-*1RA, IL-12, LINE-1, MAOA, Q6NUR6, RELN* (rs2229864), *RTL*, *SHANK3*, *TAMRTKS*, and *VDR*. The first study that used sequencing in Africa was conducted in the Egyptian population, linking the *DRD1* (rs453) variant to autism (Azzam et al., 2018). The *COMT* Val58Met polymorphism and ASD in Egyptian young people were also linked (Karam et al., 2013). A case-control study performed by Egyptian researchers revealed strong evidence that the DRD4 7/7 allele could be associated with an increased risk of autism (Kamal et al., 2015).

Additionally, three genes involved in the tryptophan metabolic pathways—*MAOA*, *HAAO*, and *AADAT*—were found to have lower expression levels in patients with ASD, which was correlated with the severity of autism in a comparison between children with ASD, learning-disabled children, and healthy controls (Higazi et al., 2021). The *DRD2* A1+ genotype enhanced autism risk in Egypt’s autistic community according to a study on the relationship between autism and genetic variants in several neurotransmitter-related genes such as *MAOA*, *MAOB* rs1799836, and *DRD2*. SNPs and/or mutations in miR-21 or miR-431 were not found in the patients enrolled in the study (Salem et al., 2013). A genetic study conducted on Tunisian people with ASD reported an increase of *Q6NUR6* gene expression in blood (Bayou et al., 2010), which might be linked to the cause of ASD in those populations.

The results of numerous investigations point to a correlation between complex diseases brought on by genetic variants and variations in serum protein levels in solid tissues and biofluids. Genetic effects on serum proteins may provide new perspectives on the mechanisms behind the genetics of common diseases and significant characteristics. These new perspectives could help us understand the genetics of prevalent diseases and their key characteristics. Based on the analysis of *TAMRTK*s blood levels in a sample of Egyptian children with autism, the findings revealed that their levels were upregulated and had strong positive associations with the severity of the condition (Mostafa et al., 2022). In addition, the results of the study supported the immunological etiology of ASD by demonstrating that Egyptian children with ASD had an immunological impairment in the form of an elevated serum level of IL-12, which was positively linked with the severity of autistic symptoms (Ibrahim et al., 2015).

ASD in Egyptian children was found to be associated with abnormally elevated serum levels of IL-1β and IL-1RA, and polymorphisms in the IL-1β-511 and IL-1RA genotype variants may influence the risk of ASD and be used as potential biomarkers of the disorder. These findings were strongly linked to the severity of autism and behavioral impairments (Saad et al., 2020). Evaluation of the possible involvement of *RTL* and *LINE-1* methylation as biomarkers for autism revealed a substantial lower level of methylation than controls in Egyptian autistic patients with *p* < 0.001 (Salem and Ashaat, 2023). *RTL* and *LINE*-1 methylation percentages can both be used as biomarkers for autism. Mostafa and Shehab (2010) suggested that *C4B* null allele autoimmunity in Egyptian autistic people may be related to the *C4B* null allele. In the Egyptian ASD population, GABAerigic malfunction was shown to increase apoptosis. As a result, neuronal excitement and an imbalance in the inhibitory system can be connected to GABA synaptopathies and their linkage to apoptosis, which can be used as trustworthy potential biomarkers for autism (El-Ansary et al., 2021). It is interesting to note that all South African autistic ethnic groupings have shown a highly meaningful correlation with the *S/*S variant of 5-*HTTLPR* (Arieff et al., 2010). Furthermore, the 5-*HTTLPRS* allele S allele significantly increased in Egyptian autistic children (Meguid et al., 2015). The expression of the *HTR1A* gene was shown as a potential candidate gene for ASD-related pathways in an explored sample of the Egyptian population (Yahya et al., 2019). Moreover, the 5-HT2A receptor may be involved in the development of ASD, according to a study on Egyptian children with autism (Abdelrahman et al., 2015).

The role of the *RELN* gene in autism has been elucidated by many discoveries of reduced levels of the reelin protein in the brain and plasma in patients with autism. Evidence first indicated that *RELN* rs736707 may play a role in autism in South African populations (Sharma et al., 2013). Recently, *RELN* gene polymorphism (rs2229864) has been reported as a possible contributing factor for ASD genetic susceptibility and severity in Egyptians (Abdelhady et al., 2022). Two different studies were conducted in Egyptian children with autism on HLA allele. One study found that there was a considerable probability that the HLA-DRB1 allele would be linked to a family history of autoimmunity (Mostafa et al., 2013). The HLA-DR4 alleles have been also linked to autism, according to another study conducted in Egypt (El-Hossiny et al., 2023). Children with ASD in Egypt have been shown to have *SHANK3* copy number variations, and the variation in *SHANK3* copy number revealed the function of *SHANK3* in the ASD etiology (Meguid et al., 2020). One potential mechanism underpinning the etiology of ASD is gene–environment interaction. The most common type of null *GSTM1* and *GSTT1* genotypes in ASD were reported in a gene-environment interaction Egyptian study. That genotype may make autistic children more susceptible to reduced antioxidant status (GST enzyme activity), which could result in improper aluminum detoxification (Said et al., 2021).

Even though most of the results from genetics studies showed a positive association with ASD, some studies have a negative association with autism in the African population. A PCR-amplified DNA *MTHFR* gene assay revealed that the homozygous 677TT genotype was found in 23% of the children with ASD, whereas the heterozygous 677CT genotype was found in 56% of the ASD group in an Egyptian study on the MTHFR C677T polymorphism. Despite the fact that the 677CT variant alleles were increased in autistic patients, it is doubtful that these alleles cause the wide range of symptoms associated with autism (Shawky et al., 2014). Another Egyptian study analyzed a functional polymorphism of the *HFE* gene and *SLC40A1*, and the results highlighted the rarity of this functional polymorphism (Gebril and Meguid, 2011; Na and Oh, 2014). The study of two genes, *HoxA1* and *HoxB1,* appeared to be implicated in the development of autism in various groups. Nevertheless, a study could not find a statistically significant link between the prevalence of autistic children in Egypt and the variations of these two genes (Sheikh et al., 2007). ASD patients have considerably lower levels of reduced glutathione, according to an evaluation of the serum levels of oxidative stress indicators and an analysis of genetic variants of glutathione S-transferase related with autism for children with autism in Nigeria. However, in this study, the distribution of the three polymorphisms GST1, GSTM1, and GSTP1 was not linked to autism (Oshodi et al., 2017).

#### 3.1.4 Other molecular biology techniques

A total of 12 genes was identified through methylation analysis and microarray-based comparative genomic hybridization (aCGH): *CELF4, CHRFAM7A, ERICH1*, *FTHL17*, *NEXMIF, NLGN4X, PCCB, PCDHA12, PRKN*, *SPN, SYCE3, UQCRC2* and one *de novo* balanced (7; 16) (p22.1; p16.2) chromosomal aberration by cytogenetics and fluorescent *in situ* hybridization (FISH). Through methylation analysis, the results from the South African ASD cohort, both case and control *PCCB* and *PCDHA12*, had methylation levels that ranged widely: 9%–49% and 0%–54%, respectively. The *PCCB* revealed three CpG loci that were differently methylated with *p* ≤ 0.05; however, *PCDHA12* revealed two CpG sites with a significant difference between ASD and control (*p* ≤ 0.001) (Stathopoulos et al., 2020). In addition, lead, aluminum, and mercury were found at a higher concentrations in the hair of autistic children than in control children. Autism may be caused by exposure to these harmful heavy metals in the environment at critical developmental stages, as shown in the Egyptian study that used the hydride generation technique (Mohamed et al., 2015). The aCGH technology presented high pathogenicity scores in numerous genes, such as *PRKN*, *CHRFAM7A*, *SYCE3*, *UQCRC2*, *SPN*, *FTHL17*, *NLGN4X*, *ERICH1*, *NEXMIF*, and *CELF4*, in Tunisia children with ASD (Chehbani et al., 2022), and were pathogenic/likely pathogenic to ASD, as showed in this cohort study. A case report in Tunisia showed particular chromosomal abnormality in a person with autism, with *de novo* balanced (7; 16) (p22.1; p16.2) translocation (Bayou et al., 2008).

## 4 Discussion

ASD can be caused by both inherited and *de novo* gene mutations due to its extreme genetic heterogeneity. A staggering number of newly emerging mutations linked to ASD have been discovered due to advances in human genetics and sequencing technologies. The genetic causes of ASD are now better understood because of recent large-scale multinational research projects. To date, there are more than 100 genes linked to autism (Sanders et al., 2015; Grove et al., 2019; Forrest and Penzes, 2020). However, this study has found 61 genes associated with ASD in the African population. ASD-causing genes were found in nine African countries.

The majority of the studies were conducted in Egypt and South-Africa, as their governments acknowledge the importance of science and research in improving people’s standard of living and generating income for their citizens (Hardy et al., 2008; Bond et al., 2012). This has enabled them to establish genetic and genomic facilities while other countries lack an enabling environment for genomic studies, have scarce or no funding, and have complicating political contexts (Omotoso et al., 2022).

Except for GWAS, most research methods used globally for genetic studies on ASD have been applied in Africa. Our findings demonstrate that numerous genetic variations may play a role in the genetic basis of ASD in the African population. Study findings have shown that several chromosomal regions include one or more susceptibility genes for autism (Monaco and Bailey, 2001).

### 4.1 Advances in genomics for ASD analysis

The quest for the genes underlying prevalent human diseases and associated quantitative features is being revolutionized by GWAS. This method combines a thorough and objective examination of the genome with the capability of finding common alleles with few phenotypic effects (Hirschhorn and Daly, 2005). The effectiveness of this technique depends on the underlying impact sizes carried by the genuine risk variations as well as the statistical power to detect these effects given the sample size and study design. Common genetic variation accounts for around half of ASD genetic risk (Glasson et al., 2004), making GWAS an effective method for locating risk variants. Many loci linked to ASD have been discovered in non-African population using GWAS. Increased number of SNPs (iPSYCH-SSI-Broad Autism Group et al., 2016; Grove et al., 2019) and other types of variation (such as copy number variations and rare structural variants) (De La Torre-Ubieta et al., 2016) that are linked to ASD have been found during the past 10 years through GWAS. According to a meta-analysis of GWAS, there is a strong genetic correlation between ASD and a number of genes relevant to neurodevelopment, including *EXT1*, *ASTN2*, *MACROD2*, and *HDAC4* (Anney et al., 2017). In addition, it has been shown through GWAS that traits resembling autism are associated with numerous immune-related genes such as *RNF114*, *CDKN2A*, *KAZN*, *SPATA2*, and *ZNF816A* (Arenella et al., 2022). However, GWAS must have a sample size with enough statistical power to identify the genes responsible for human complex disorders (Baranger et al., 2023). Thus, care must be taken in study design, execution, analysis, and interpretation to minimize the drawbacks of this strategy.

Most studies have focused on populations from the United States, Europe, and Asia, and there is little information regarding the genetic composition of ASD across Africa. This scoping review notes that no gene has been found in the African population using GWAS. According to the African Genome Variation Project, the population of Ethiopia has the highest percentage of unique and private genomic diversity in all of Africa, accounting for about 24% of genomic variants (Gurdasani et al., 2015). It represents the highest prevalence of ASD for an East African population (Barnevik-Olsson et al., 2008; Magnusson et al., 2012). Although the prevalence rate of ASD is higher in East African populations than the rest of the globe, no prevalence study has been carried out in East Africa’s nations. Numerous studies carried out in Europe for immigrant populations highlight a higher frequency of ASD among offspring born to parents from East Africa (Barnevik-Olsson et al., 2008; Magnusson et al., 2012). Thus, large-scale GWAS studies are required in Africa to uncover many genetic variants linked to ASD because the continent still retains much of the diversity found in the human genome. Understanding common biological processes that influence health and disease in all groups will result from identifying genetic variations in African populations.

The use of sequencing technology for disease-causing gene mutation discovery has the advantage of emphasizing the importance of this discovery for proper patient management and family counseling (Lohmann and Klein, 2014). The patient’s entire exome or genome may be precisely analyzed using next-generation sequencing and can be found in a single experiment (Gupta and Verma, 2019). Sequencing technology is especially well-suited for studying the genetics of heterogeneous traits like ASD; it has discovered a meaningful number of arising mutations linked to ASD. The ability of next-generation sequencing to find risk-mediating variations in single families or small cohorts of ASD patients was demonstrated in a number of early studies (O’Roak et al., 2012; Sanders et al., 2012). To discover ASD risk genes in the coding regions of human DNA and search for *de novo* mutations from various populations, several researchers have utilized a variety of sequencing methodologies, such as whole genome sequencing (WGS) and whole exome sequencing (WES) (Michaelson et al., 2012; Neale et al., 2012). Although sequencing technology has only recently been adopted in several African nations, it has allowed researchers to identify numerous genes associated with ASD. Common and rare variants of the voltage-gated calcium channel gene *CACNA1C* have been associated with autism and neurodevelopmental disorders, including schizophrenia, bipolar disorder, and attention deficit hyperactivity disorder (ADHD) (Buddell et al., 2019). *FMR1*, the gene linked to fragile X syndrome (FXS), is located on chromosome X, and an unstable CGG repeat in the 5' untranslated region of the gene prevents *FMR1* from functioning properly, resulting in FXS. One of the primary gene regulators in the central nervous system (CNS) is FMRP, the protein that results from *FMR1*. It has been discovered that individuals with ASD who do not have FXS have defective multiple FMRP-regulated pathways (Fyke and Velinov, 2021). The two most commonly identified mutations in ASD patients, *CACNA1C* and *FMR1* (Tuncay et al., 2023), were also found in the East African population. A *CHD7* intronic variant causes some important genes linked to neural developmental disorders to become dysregulated; this effect is comparable to that brought on by gene deletion (Zhang et al., 2021). The *CHD7* intronic variation is potentially an autism susceptibility locus within the Chinese ASD population (Zhang et al., 2021) and has also been identified in the East African cohort (Tuncay et al., 2023).

In addition, a mediator of canonical Wnt signaling, *TCF7L2*, is recognized to be crucial for the development of the central nervous system (CNS). Heterozygous *de novo* mutations in *TCF7L2* exhibit neurodevelopmental symptoms (Dias et al., 2021). Previously, *TCF7L2* reported in non-African populations (Dias et al., 2021) was also found in East Africa (Tuncay et al., 2023). Protocadherin α (PCDHA) is involved in serotonergic innervation of the brain and synaptic specificity (Katori et al., 2009), making it a good candidate gene for autism. Strong genetic evidence has been reported of the possible role of *PCDHA* as an autism candidate gene in the Japanese population (Anitha et al., 2013), and the mutation in one *PCDHA* family gene, *PCDHAC1*, was also found in East Africa (Tuncay et al., 2023). A 2-methylbutyryl-CoA dehydrogenase deficit, sometimes referred to as “short/branched chain acyl-CoA dehydrogenase deficiency” (SBCADD), is caused by a malfunction in the breakdown process of the amino acid L-isoleucine. Isoleucine is oxidized and used as fuel in the brain. Two cases from Somalia and Eritrea displayed mutated *SBCADD* gene (Kanavin et al., 2007). Major transcription factors encoded by FEZF2 modulate neuron location and identity. *FEZF2* and ASD have been genetically linked in two sizable European ancestry cohorts, and this relationship has been confirmed in two more cohorts (Wang et al., 2009). Interestingly, the *FEZF2* gene was also found in the Moroccan population (Bensaid et al., 2019).

The scaffolding proteins found in glutamatergic synapses’ postsynaptic density (PSD) encoded by the *SHANK2* and *SHANK3* genes, and *de novo SHANK2* deletions confirmed in Caucasian ancestry ASD patients (Leblond et al., 2012), were also reported in a patient of Ethiopian ancestry (Lu et al., 2018). Furthermore, *SHANK3* deletion was found in Chinese people with ASD through a genome-wide copy number variation analysis (Guo et al., 2017); *SHANK3* dose contributes to the pathogenesis of ASD for Egyptian autistic children (Meguid et al., 2020). There is mounting proof that DNA methylation influences ASD characteristics. Retinal degeneration and progressive renal failure are potential outcomes of AHI1 mutations (Parisi et al., 2006), and GLRA2 was found to be a new gene that causes high myopia (Tian et al., 2023). Through whole-genome DNA methylation screening, *AHI1* and *GLRA2* genes were differentially methylated in a South African cohort with ASD (Stathopoulos et al., 2018), and a United States study provided evidence for an associated haplotype in *AHI1* with ASD (Alvarez Retuerto et al., 2008). Moreover, *GLRA2* was reported in autistic people from France (Pilorge et al., 2016). The ubiquitin-proteasome pathway that may be responsible for the downregulation of SET domain-containing 5 (SETD5) is often overactive and mutated in human NDDs as well as cancer (Li et al., 2023). The ASD-linked genes *SETD5* (Grozeva et al., 2014) and *MTR* (Muratore et al., 2013), which are directly involved in DNA methylation, were differentially methylated in the ASD South African population (Stathopoulos et al., 2018). Peroxisome proliferator-activated receptor-γcoactivator (PGC-1α) is involved in remodeling muscle tissue into a fiber-like composition, and it increases mitochondrial biogenesis (Liang and Ward, 2006). The differential methylation of the *PGC*-1*α* gene was reported in the ASD South-African population (Bam et al., 2021), and a Chinese Han study also suggested that the SIRT1/PGC-1α signaling pathway has a crucial function in patients with ASD (Bu et al., 2017). In addition, the optic atrophy 1 (*OPA1*) gene, involved in mitochondrial fusion, works in conjunction with stomatin-like protein 2 (*STOML2*) (Tondera et al., 2009). Study results for those two genes in a South African population with ASD showed that the *OPA1* and *STOML2* genes were significantly differentially methylated, which may implicate their contribution in ASD etiology (Bam et al., 2021). Furthermore, in a Chilean study, autistic children had significantly higher levels of mtDNA in their oral mucosa, and there was an expression of *MFN2, FIS1*, and *OPA1* involved in the fission/fusion processes of mitochondria (Carrasco et al., 2019). Several co-occurring symptoms linked to *SATB2* gene variations have led to identifying a single clinically recognized syndrome known as SATB2-associated syndrome (SAS) (Döcker et al., 2014). There was a decreased metabolic response to tryptophan for SAS patients from United States, and that has been associated with ASD (Boccuto et al., 2013). The *SATB2* gene has also been found in Moroccan people (Bensaid et al., 2019). Furthermore, *TSHZ3* encoding the zinc-finger homeodomain transcription factor TSHZ3 that was found in networks of human neocortical genes is strongly expressed throughout late fetal development and is implicated in neurodevelopmental and neuropsychiatric diseases (Caubit et al., 2016; Li M. et al., 2018). This gene linked to ASD in a Moroccan study (Bensaid et al., 2019) was identified as one of the ASD-related genes differently expressed in the brain in a Chinese study (Li et al., 2020). Additionally, *TBR1*, a T-box transcription factor (TF) essential for controlling cortical development (Han et al., 2011), previously reported as ASD risk contributing mutation (O’Roak et al., 2012) in a United States study, was also identified in Moroccan ASD families (Bensaid et al., 2019). Protein coding gene TBC1 domain family member 8 (*TBC1D8*) is a new ASD gene found in Libyan siblings with ASD (Zeglam and Alhmadi, 2020) but not reported in any other populations.

Re-sequencing the complete genome in patients and controls to look for a variant or group of variants enriched or depleted in disease cases yields the most thorough study of candidate genes. However, because such research is still time-consuming and expensive, it has mostly focused on the coding areas of a small number of potential genes. In addition to revealing complex inheritance patterns, such as the inheritance of two autosomal recessive gene mutations in the same patient and family, the use of sequencing technology for disease-causing gene mutation discovery has the advantage of emphasizing the importance of this discovery for proper patient management and family counseling (Lohmann and Klein, 2014). The patient’s entire exome or genome may be precisely analyzed using next-generation sequencing, and single nucleotide variations can be found in a single experiment (Gupta and Verma, 2019). As the most advanced molecular approach available today, PCR technology has a wide range of present and future therapeutic uses, including pathogen detection, assessment of emerging novel infections, surveillance, early detection of biothreat agents, and antibiotic resistance profiling (Yang and Rothman, 2004). PCR is used by a variety of researchers to analyze mutations that arise in many genetic disorders (Reiss and Cooper, 1990). PCR-based methods were mostly adopted in Africa for studying the genetic etiology of ASD. The reelin gene (RELN) plays a key role in the brain’s regulation of neuronal migration, synaptogenesis, and synaptic plasticity. Therefore, the RELN signaling pathway has been linked to a number of neurological conditions in humans, including mental retardation, ASD, and schizophrenia (Jossin, 2020). Two different RELN SNPs previously linked to ASD in non-African population were also identified in Africa. One RELN SNP, rs736707, discovered to be significantly linked to ASD in Asian groups (Chen et al., 2017), was reported as an ASD risk factor for the South African population (Sharma et al., 2013). Another RELN SNP, rs2229864, associated with the genetic predisposition for ASD in the Chinese Han population (Wang et al., 2018), was also found to be related to ASD in Egyptian children (Abdelhady et al., 2022). Brain serotonin (5-HT) is widely recognized to be involved in the regulation of both normal and abnormal behavior (Blier and Ward, 2003; Ögren et al., 2008). Numerous studies have suggested that the neurobiological process underlying ASD is likely to involve function-impairing polymorphisms of the 5-HT system, including 5-*HTTLPR*, 5-*HTR1A*, and 5-*HTR2A* (Chugani, 2002; Richardson-Jones et al., 2010). Strong associations have been found between the 5-*HTTLPR* polymorphism and various autistic populations worldwide, according to numerous case-control studies. In a United States study, a significant relationship was found between ASD and the serotonin transporter gene promotor polymorphism (*5-HTTLPR*) (Brune et al., 2006), and was also found in all ASD patients in South Africa (Arieff et al., 2010). Moreover, the *5-HTTLPRS* allele was significantly increased in Egyptian autistic children (Meguid et al., 2015). Nevertheless, an association between the 5-HTTLPR polymorphism and autism has not been found by a meta-analysis (Wang et al., 2019). Moreover, the *HT2A* gene reported in the Croatian (Hranilovic et al., 2010) and Korean (Cho et al., 2007) populations was also shown in Egyptian autistic children (Abdelrahman et al., 2015). The steroid hormone vitamin D is well known for its function in neuronal growth and neuroprotection. Consequently, deficiencies in the vitamin D pathway might contribute to the development of ASD (Zhang et al., 2018). It has been demonstrated that some *VDR* gene polymorphisms, particularly rs731276, are a risk factor for childhood ASD in the Chinese Han community (Zhang et al., 2018) and are linked to ASD in the Egyptian population (Arafa et al., 2021).

Autoimmunity and various autoimmune disorders may be influenced by complement (C) 4B null alleles, which result in low levels of C4B protein. The *C4B* null allele had a high risk of association with autism, and there is a connection between the *C4B* null allele and a family history of autoimmunity (Mostafa and Shehab, 2010). Therefore, autoimmunity in Egyptian patients with ASD may be significantly influenced by the *C4B* null allele (Mostafa and Shehab, 2010), and the US study’s findings indicate that this allele is significantly linked to an increased risk of autism (Odell et al., 2005). The tryptophan metabolism-related genes such as aminoadipate aminotransferase (*AADAT*), 3-hydroxyanthranilate oxygenase (*HAAO*), and monoamine oxygenase A (*MAOA*) were found in children with ASD in Egypt (Higazi et al., 2021). Interestingly, *de novo* missense mutation in *AADAT* found in Chinese families with ASD (Li S.-J. et al., 2018) was suggested as the basic etiology of ASD for those families. ASD patients in the United States have shown decreased expression levels of the genes *AADAT*, *HAAO*, and *MAOA* (Boccuto et al., 2013). In addition, the enzyme catechol-O-methyltransferase (COMT) has been linked to aberrant dopaminergic activity, suggesting that this gene may be involved in the etiology of ASD (Esmaiel et al., 2020). The *COMT* variant is linked to hyperactivity symptoms in Egyptian children with ASD (Karam et al., 2013), and findings from a United States study suggested that the ASD phenotype may be influenced by the *COMT* gene (Radoeva et al., 2014).

Dopamine is crucial for maintaining appropriate attention. Hence, research has examined the dopaminergic system’s genes, particularly concerning attention problems. The *DRD1* and *DRD2* genes were identified in Egyptian ASD autistic patients (Salem et al., 2013; Azzam et al., 2018), and according to the results from an Italian study, both *DRD1* and *DRD2* receptor SNPs may be regarded as possible risk factors for ASD (Mariggiò et al., 2021). The human dopamine receptor D4 (*DRD4*) gene, found on chromosome 11p close to the telomere, has an unusually high level of expressed polymorphism. A clinically higher risk for autistic symptoms may be associated with the DRD4 7-repeat allele (Reiersen and Todorov, 2011) according to US findings from Missouri-born twins study, which are similar to the findings of another Egyptian study (Kamal et al., 2015). In the adult human brain, gamma-aminobutyric acid (GABA) is the main inhibitory neurotransmitter. Several neuropsychiatric disorders, including, anxiety, epilepsy, and learning disabilities, have all been linked to GABAergic dysfunction (Coghlan et al., 2012). In a US study, autistic children exhibited considerably lower levels of sensorimotor *GABA* than healthy controls (Puts et al., 2017), and that was also found in an Egyptian study (El-Ansary et al., 2021). HLA proteins are involved in the development of the central nervous system (CNS), cerebral hemisphere specialization, synaptic function, and neural cell interactions (Torres et al., 2012), and several HLA alleles, including HLA-DRB1 alleles, are linked to ASD (Polleux and Lauder, 2004). Findings from a Han Chinese study suggest that the *HLA*-*DRB1* gene is linked to ASD (Chien et al., 2012), which has been reported in Egypt (Mostafa et al., 2013). Furthermore, a US study suggested that *HLA*-*DR4* is linked to ASD (Torres et al., 2002), where autistic patients demonstrated high HLA DR4 allele frequency, and an Egyptian study has also reported such results (El-Hossiny et al., 2023). In addition, long interspersed nucleotide element-1 (LINE-1) is a type of non-long terminal repeat retrotransposon that has been linked to disorders including ASD due to aberrant DNAm (Saeliw et al., 2018). LINE-1 methylation was shown to be substantially lower in lymphoblastoid cell lines from ASD patients in Italy (Tangsuwansri et al., 2018), and a recent study in Egypt has reported similar results (Salem and Ashaat, 2023). Furthermore, the gene retrotransposon Gag-like 1 (RTL1), a paternally expressed gene, is crucial for placenta maintenance (Sekita et al., 2008). The *RTL* gene also reported in this study was found in the Iranian population, and the findings showed a sexually dimorphic pattern of *RTL* in children with ASD (Panahi et al., 2023).

Microarray-based comparative genomic hybridization aCGH technology performed on Tunisian children diagnosed with ASD has reported numerous pathogenic or likely pathogenic variants such as *CELF4, CHRFAM7A, ERICH1, FTHL17, NEXMIF, NLGN4X, PRKN, SPN, SYCE3,* and *UQCRC2* (Chehbani et al., 2022). Several of these were reported to be associated with ASD worldwide, such as both *CELF4* and *CHRFAM7A* in Italy (Bacchelli et al., 2015; Barone et al., 2017), *NEXMIF* in Switzerland (Lambert et al., 2018), and both *NLGN4X* and *UQCRC2* in China (Yu et al., 2011; Shen et al., 2019). The ASD etiology is thought to be caused by epigenetic processes like DNA methylation, RNA interference, and chromatin alteration (Tremblay and Jiang, 2019). The methylation-dependent regulation of transcription has become an intriguing theory for the etiology of ASD because of next-generation sequencing and the identification of DNA alterations. The *PCCB* gene mutations found in ASD patients in Belgium (Witters et al., 2016) were differentially hypomethylated in South-Africa patients affected with ASD (Stathopoulos et al., 2020). Another gene, *PCDHA12*, reported in that South African study was also studied in Japan, providing strong genetic evidence that *PCDHA12* may be a viable candidate gene for autism (Anitha et al., 2013). Moreover, genetic and environment interactions have also been identified as potential factors contributing to ASD. Many hazardous metals must be detoxified, and glutathione-S-transferase (*GST*) genes and associated enzymes are essential. The study findings imply that among Jamaican children with ASD, the existence of active versions of the *GSTT1* and *GSTM1* genes may be linked to a higher ability for lead (Pb) detoxification (Rahbar et al., 2022), and an Egyptian study also found that the most prevalent genotype associated with ASD, null GSTM1 and GSTT1, may predispose ASD children to a lower antioxidant status (Said et al., 2021), which can ultimately result in improper aluminum detoxification. There is significantly more aluminum in the hair of children diagnosed with ASD, and the development of autism in these children may be significantly influenced by oxidative indicators that result in oxidative damage (Said et al., 2021). However, a Nigerian study reported opposite results on GST genes by discovering that although ASD patients had significantly lower levels of reduced glutathione, there was no correlation between ASD and the distribution of the *GSTT1*, *GSTM1*, or *GSTP1* polymorphisms (Oshodi et al., 2017). Furthermore, the limited sample size made it difficult to generalize these findings to the whole population. Large sample sizes are typically required in case-control studies to detect such effects because common diseases are caused by complicated interactions among several genetic variations and environmental risk factors (Moonesinghe et al., 2008). In addition, several other genes such as *DISC1*, *GABRB3*, *GSTP1, HFE*, *HOXA1*/*HOXB1, SLC40A1*, and *MTHFR* 677C>T were unrelated to risk of ASD in Africa. Numerous chromosomal abnormalities, both balanced and unbalanced, have been linked to autism (Wassink et al., 2001; The Autism Genome Project Consortium, 2007), and *de novo* balanced (7; 16) (p22.1; p16.2) translocation was reported in a Tunisian male patient with autism (Bayou et al., 2008). According to our findings, a total of 12 genes associated with ASD in Africa—*ERICH1, FTHL17, GABPA, IL-1β-511* and *IL-1RA, IL-12, PRKN, Q6NUR6, SPN, STOML2, SYCE3*, *TAMRTKS*, and *TBC1D8*—were not identified in non-African populations.

On other hand, 20 ASD genes, each reported at least in two population studies, were identified in non-African populations that are yet to be confirmed/reported in Africa, perhaps because molecular research on ASD has continually underrepresented African populations, and the majority of current research on ASD is carried out in high-income countries (Frickel et al., 2023). Acetylserotonin methyltransferase (*ASMT*), a component of the final stage of melatonin production, was reported in China (Wang et al., 2013; Jonsson et al., 2014). The chromodomain helicase DNA binding protein 8 (*CHD8*) interacting with beta-catenin for chromatin remodeling (Bernier et al., 2014) was reported in the United States. Contactin-associated protein-like 2 (*CNTNAP2*), a member of the neurexin superfamily that plays a crucial role in brain development (Li et al., 2010; Nascimento et al., 2016), was reported in China and Brazil. The dopamine-3-receptor (*DRD3*) gene linked to posttraumatic stress disorder-related impairments in emotion reactivity, executive functioning, and stress-responding (De Krom et al., 2009) was reported in the Netherlands and United Kingdom. Engrailed 2 (*EN2*), a homeobox transcription factor that plays a role in the cerebellum’s patterning during brain development (Wang et al., 2008; Sen et al., 2010) was reported in China and India. The forkhead-box protein P1(*FOXP1*) gene encoding a transcription factor crucial for the early development of numerous organ systems, including the brain (Hamdan et al., 2010; Chien et al., 2013), was reported in Taiwan and Canada. Gamma-aminobutyric acid (*GABRB3*), the primary inhibitory neurotransmitter in the brain (Buxbaum et al., 2002; Warrier et al., 2013; Chen et al., 2014), was reported in the United States, United Kingdom, and Taiwan. The glutamate-binding GluN2 (GRIN2B) involved in circuit construction, brain development, and potential cell migration and differentiation (Yoo et al., 2012; Pan et al., 2015) was reported in China and Korea. HoxA, a key player in the formation of hindbrain neural structures (Ingram et al., 2000; Conciatori et al., 2004), was reported in the United States and Italy. The methyl CpG binding protein 2 (*MECP2*) gene (Nagarajan et al., 2006; Wen et al., 2017) was reported in the United States and China. The *MET* mediating hepatocyte growth factor signaling (Campbell et al., 2009; Jackson et al., 2009) was reported in Italy and the United States. Neuroligin 3 (*NLGN3*) regulating synapse organization (Yu et al., 2011; Oleari et al., 2023) was reported in China and Italy, while Neuroligin4 (*NLGN4*) was reported in France, Bulgaria, and Japan (Laumonnier et al., 2004; Avdjieva-Tzavella et al., 2012; Toya et al., 2023). In addition, Neurexin-1 (*NRXN1)* was reported in China and the United States (Liu et al., 2012; Griswold et al., 2015). The oxytocin receptor gene (OXTR) which plays a role in social-emotional behaviors (Wu et al., 2005; Di Napoli et al., 2014) was reported in the United Kingdom and China. The patched domain containing protein 1(*PTCHD1*) was reported in Spain, the Netherlands, Germany and Italy (Torrico et al., 2015). PTEN, a PI3K/AKT pathway negative regulator, was reported in the United States (Buxbaum et al., 2007). The *SLC25A12* gene involved in catalysis of the mitochondrial carrier of aspartate-glutamate exchange (Turunen et al., 2008; Aoki and Cortese, 2016) was reported in Finland and the United Kingdom. *SRRM4*, a member of an RNA splicing factor family that controls the incorporation of specific genetic material (Irimia et al., 2014; Narita et al., 2020), was reported in Italy and Japan. Lastly, *SYNGAP1* that regulates developmental excitatory synapse formation and function was reported in Canada (Berryer et al., 2013).

### 4.2 Recommendations for genomic ASD study

Given the genetic diversity found in Africa, more extensive sequencing of samples from various African populations is lacking. There is a need to conduct sequencing research in various African countries, which could identify new genetic variants linked to ASD in Africa. The use of sequencing techniques to identify ASD risk genes and unreported mutations in well-known sites has shown potential. The potential genes that have not yet been linked to ASD will need to undergo sequencing in larger cohorts and additional experimental validation to prove causation or the genes’ role in the disorder. Most genetic studies for ASD in Africa adopted PCR-based methods; however, fewer ASD risk genes were identified through this study design than in other techniques. The PRC-RFLP method has been primarily performed in Africa, as it is faster and cheaper than sequencing methods. Sequencing is a common technique for the identification of mutations. Additionally, there is a need for many genetic studies that use sequencing technology or GWAS across various African countries, thus helping to identify a significant number of *de novo* genetic variants associated with ASD due to using different ethnic groups. Several identified studies in this paper have shown that findings can be limited by some technical difficulties and relatively small sample size due to the cost of the sample for analysis. To better understand the hundreds or thousands of common and unusual genetic variants associated with ASD in the African population, sample sizes must be significantly increased. A greater sample size should be used in subsequent efforts to enable additional studies, such as better cataloging of structural variation and improved disease prediction.

### 4.3 Clinical and therapeutic implications

ASD risk is believed to be influenced by numerous common gene variants. ASD risk genes found in Africa are linked to various human disorders (Table 1). The primary symptoms of ASD, including difficulties with social communication or repetitive activities, are not currently treated with pharmaceuticals; instead, behavioral therapy and the use of tightly regulated learning environments are used as treatments for ASD. Up until now, the US Food and Drug Administration (FDA) has only licensed and approved two medications for the treatment of irritability associated with ASD: the antipsychotics risperidone and aripiprazole (Lamy and Erickson, 2018). However, many additional pharmacological treatments have shown successful therapeutic management of ASD symptoms, including: atypical antipsychotics (risperidone, olanzapine, clozapine) for temper tantrums, aggression, or self-destructive behavior; selective serotonin reuptake inhibitors (sertraline, citalopram, fluoxetine) for anxiety and repetitive behaviors; psychostimulants (methylphenidate) for hyperactivity; opioid antagonists (naltrexone) for hyperactivity (Aman, 2004; Kumar et al., 2012). Additionally, a few of the most effective medications are currently undergoing various stages of clinical trials for the treatment of ASD’s behavioral and neurological symptoms (Eissa et al., 2018).

Precision medicine is a future medical practice based on algorithms that consider the patient’s features, such as their genome, epigenetics, and lifestyle. The main objective of ASD research is to find effective treatments for people with ASD (Fischbach et al., 2016), and the development of a customized medicinal strategy for these individuals depends on the identification of a genetic etiology (Schaefer, 2016). According to data from a Norwegian sample study, many parents of children with ASD obtain positive results from clinical genetic testing (CGT) since there may be an etiological cause (Johannessen et al., 2017). However, investigations are failing if African people and other underrepresented ethnicities are not included in genetic studies. Many unidentified genetic variants probably contribute to medically significant traits in African and non-African populations. Thus, the identification of genes associated with ASD in Africa’s population would help in the pharmaceutical production of therapeutics that would meet the genetic etiology of ASD in Africa.

### 4.4 Study limitations

The exclusion of gray studies, such as thesis projects, is a limitation given the findings of such excluded studies. However, as genetic research on ASD is not a very developed research field in Africa, theses may contribute significantly to the overall research evidence; hence, their exclusion may have resulted in an incomplete picture of the existing literature. Furthermore, our findings reveal a limitation of small sample sizes in the identified studies, which could account for the discrepancies in the results; thus, future research should focus on using larger samples from multiple sites to generalize its findings more broadly.

## 5 Conclusion

Our scoping review examined research conducted in Africa on genetic factors potentially associated with ASD, categorized into groups based on study methodology: GWAS, sequencing, PCR-based methods, and other techniques. We found that PCR-based study design is the most performed in Africa. However, only a limited number of ASD risk genes and polymorphisms were reported using that method. Even though sequencing performed in various African countries is limited, many genetic variants associated with ASD were identified through this approach. However, no study identified any ASD gene with GWAS, even though it has been used in non-African countries. Furthermore, larger samples should be used in future studies to enable additional analysis, such as better cataloging of structural variation. As these are developing countries, grants for research collaboration should strengthen research capacity in Africa. African ASD could be highly attributable to genetic factors, as shown in the results of the few studies that have been conducted. Large-scale research on the genetics and prevalence of ASD is required in Africa, which will support increasing efforts to understand the genetic causes of ASD and other NDDs in the continent’s population and thus assist researchers and pharmaceutical industries to propose therapies that will meet the needs of African populations.
